# Morphine compromises bronchial epithelial TLR2/IL17R signaling crosstalk, necessary for lung IL17 homeostasis

**DOI:** 10.1038/srep11384

**Published:** 2015-06-15

**Authors:** Santanu Banerjee, Jana Ninkovic, Jingjing Meng, Umakant Sharma, Jing Ma, Richard Charboneau, Sabita Roy

**Affiliations:** 1 Surgery, Basic and Translational Research, University of Minnesota, 515 Delaware St SE, Minneapolis, MN, USA; 2 Pharmacology, University of Minnesota, 321 Church St SE, Minneapolis, MN, USA

## Abstract

Opportunistic lung infection and inflammation is a hallmark of chronic recreational/clinical use of morphine. We show that early induction of IL17 from the bronchial epithelium, following pathogenic encounter is a protective response, which contributes to pathogenic clearance and currently attributed to TLR2 activation in immune cells. Concurrent activation of TLR2 and IL17R in bronchial epithelium results in the sequestration of MyD88 (TLR2 adapter) by Act1/CIKS (IL17R adapter), thereby turning off TLR2 signaling to restore homeostasis. Morphine inhibits the early IL17 release and interaction between Act1 and MyD88, leading to decreased pathogenic clearance and sustained inflammation. Hence, we propose that therapeutically targeting either TLR2 or IL17 in bronchial epithelia, in the context of morphine, can restore inflammatory homeostasis.

Imbalance in the early protective inflammatory response against opportunistic infection results in pathogenic supremacy and uncontrolled inflammation, eventually leading to defective innate/adaptive response and host tissue damage^1^. Early and effective innate response to pathogenic insult is the key to a robust adaptive immune programming and memory^2^. Any disruption in the innate response cascade would, by association, result in a defective adaptive response, increased pathogenic loads and chronic inflammation^1^,^3^. Pulmonary inflammation, due to a variety of direct or indirect insults, pathogenic or otherwise, result in impaired innate and adaptive immune responses and presents major co-morbidities and yet, is one of the most understudied area in disease control and health^1^,^4^.

Morphine and its derivatives are one of the most abused recreational drugs as well as the most effective analgesic in post-operative pain management^3^,^5^. A whole body of literature describes the adverse effects of morphine on the immune system in the context of medical or recreational use of the drug (for review, see Roy *et al.*, 2011)^6^. In terms of lung inflammation, morphine has been implicated in inhibition of chemo- and inflation reflexes^7^ and inducing acute lung injury in human^8^. High systemic concentration of morphine in rodents and human has been shown to increase susceptibility to opportunistic infections in lungs, reduce pathogenic clearance, induce chronic inflammation and increase bacterial translocation across mucosal surfaces^3^,^6^,^9^,^10^,^11^,^12^,^13^, leading to sepsis and septic shock^6^,^11^,^14^. In most cases, as we have shown before, morphine induced chronic inflammation could be attributed to initial immunosuppression, mucosal barrier compromise, bacterial translocation across mucosa, toll-like receptor stimulation, culminating in chronic inflammation, likely associated with impaired clearance due to morphine-induced reduction of macrophage-mediated phagocytosis^3^,^10^,^11^,^12^,^13^,^15^,^16^. Coming from a variety of organs and cell-types, these studies still do not present a focused perspective on the mechanisms involved in lung inflammation and morphine mediated inhibition of its resolution.

Pulmonary inflammation due to infection (mostly *pneumococcal*) or auto-immune pre-dispositions (e.g. cystic fibrosis) have recently been attributed to the modulation of interleukin-17A (IL-17) levels in the lungs^17^,^18^,^19^. Early induction of IL-17 following pathogenic encounter has been shown in these studies to be a protective response against pathogens in the lungs, which has so far been attributed to toll-like receptor (TLR)-2 activation in the circulating immune cells^18^,^20^. Recently, by way of a paradigm shift, lung epithelial cells have been reported to be capable of mounting its own innate immune response^21^,^22^,^23^, independent of the circulating immune cells, but dependent on myeloid differentiation factor (MyD)-88^24^. We have shown earlier that TLR stimulation can induce IL-17 production in lungs as early as 4 hours, which results in neutrophil recruitment and subsequent downregulation of IL-17 release, which is subdued by chronic morphine^10^,^12^. In this study, we re-establish the immune-cell independent capability of the bronchial epithelial cells in mounting a very early IL-17 response to *pneumococcus* infection. We show for the first time that the bronchial epithelial cells have the requisite components to synthesize IL-17 and the early release upon *pneumococcal* stimulation is independent of *de novo* synthesis. We demonstrate for the first time that the IL-17 release is TLR-2 (and not TLR-4) dependent and the TLR-2 and IL-17 receptor (IL-17R) mediated pathways cross-talk with each other to foster IL-17 homeostasis in the lungs after an initial surge. We show that chronic morphine disrupts the cross-talk between the pathways and induces chronic inflammation in the lungs. Since an early cytokine response to the pathogenic insult constitutes the first step in the cascade leading to a robust adaptive immune response and immune memory, morphine modulation of this step invariably results in defective downstream responses as demonstrated earlier^3^,^8^,^11^,^12^,^13^,^15^,^25^,^26^. Finally, we demonstrate the dual effect of IL-17 on the mucosal barrier and its role in neutrophil recruitment. We show how morphine modulates both to prevent the lung to reach a steady state after an initial cytokine response.

A greater understanding of the enhanced role of bronchial epithelium in serving as an active immune barrier, in addition to its established role as a passive one, opens up new opportunities in the treatment strategies for various cancers and HIV infection. Specially in lung inflammation, where treatment strategies or infection result in immune compromise^22^, since it provides a larger surface area as therapeutic target with lesser turnover rate^24^ in immune compromised patients.

## Results

### TLR2 stimulation leads to early IL17 accumulation in the Broncheo-Alveolar Lavage (BAL), which is suppressed by morphine

**Figure 1:**
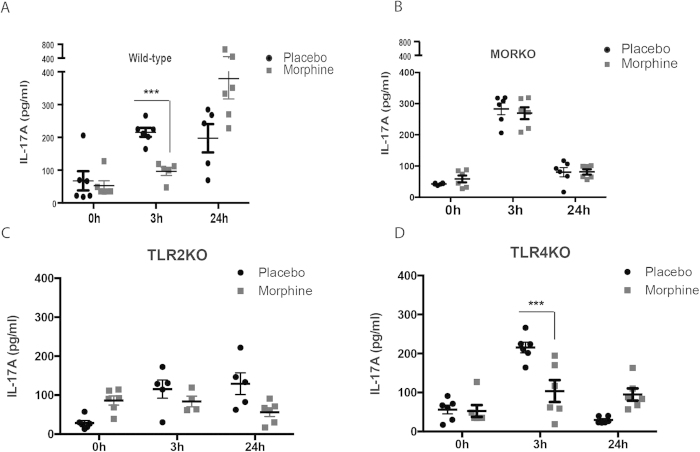
Morphine modulates TLR2 mediated IL-17 release in the lungs. Mice implanted with placebo or 75 mg slow-release morphine pellets (24 hours) were intra-nasally administered with *pneumococcus* lysate (PL) for 3 and 24 hours. Controls were maintained for 0 hours/no PL. At specified time-points, batches of animals (n = 5 or 6) were sacrificed and broncheo-alveolar lavage (BAL) was harvested using tracheal catheterization. IL-17 levels in the BAL of (**a**) Wild-type C57Bl6/j, (**b**) μ-opioid receptor knockout, (**c**) TLR2 knockout and (**d**) TLR4 knockout mice were determined using ELISA. (Error bars are SEM, ***p < 0.05 between placebo and morphine implanted animals within the same time-point).

Since IL-17A is one of the most significant contributors of lung inflammation due to opportunistic infections^12^, we set out to see the duration of IL-17A response and the effects of morphine on this phenomenon. Wild-type (C57Bl/6j) or μ-opioid receptor knockout (MORKO) mice were implanted with placebo or 75 mg slow release morphine pellets (NIDA) subcutaneously. Live *Pneumococcus* (10^7^ cfu/animal) or *Pneumococcal* lysate (100 μg/animal) was intra-nasally administered, 24 hours post placebo/morphine pellet implantation. Batches of animals were sacrificed at 3 h and 24 h time-points, with 0h/no *Pneumococcal* lysate group as control. BAL fluid was harvested by tracheal catheterization and IL-17A levels measured by ELISA (ebiosciences). Results show a release of IL-17A into the BAL as peaking at 3 hours post-TLR stimulation in WT mice, which is maintained till 24 hours. In morphine implanted WT mice, IL-17A release is significantly blunted at 3hours and this level is maintained at the 24 hours, which then is significantly higher than the placebo group at the same time-point (Fig. 1A). MORKO mice, on the other hand, exhibited a robust release of IL17A at 3hours and near-basal levels at 24hours for both placebo and morphine implanted animals (Fig. 1B). Since *Pneumococcus* primarily stimulates TLR2 and to some extent, TLR4^10^,^12^, we applied the stimulation model to TLR2 knockout mice (TLR2KO; Fig. 1C) and show a severely muted IL-17A response in the BAL. TLR4 knockout animals (TLR4KO), however, exhibit a slightly muted, yet similar IL-17A response to the WT animals (Fig. 1D).

### Bronchial epithelial cells have the machinery and capability to constitutively express IL-17A

**Figure 2:**
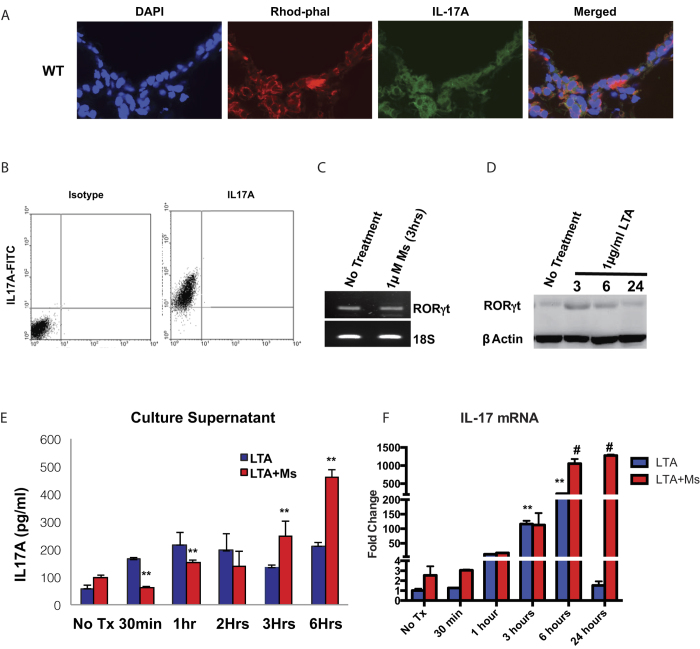
Bronchial epithelial cells have the requisite machinery to synthesize and maintain a pool of pre-made IL-17, which is released upon TLR2 stimulation. (**a**) WT; C57Bl6/j mice were sacrificed and 5 μm lung cryo-sections were stained for nucleus (DAPI), F-actin (rhodamine-phalloidin) and IL-17 (anti-IL-17 antibody-FITC). Figure representative of n = 4. Human cell-line, 16HBE14o was used to study the specific role of the bronchial epithelium in IL-17 production and release. (**b**) Flow-cytometric detection of IL-17 in untreated epithelial cells with corresponding isotype control. (**c**) 16HBE14o cells were treated with saline or 1 μM morphine for indicated time point and RORγt total mRNA levels were studied with 18 s rRNA as loading control. No differences were seen between saline and morphine treatment. (**d**) Western blot analysis of RORγt protein levels from 16HBE14o cells, treated with TLR2-specific Lipotechoic acid (LTA) for 3, 6 and 24 hours with ß-actin as load control. (**e**) ELISA quantitation of IL-17 levels, released from the 16HBE14o cells with LTA stimulation for 0.5, 1, 2, 3 and 6 hours and morphine modulation of the same. [Data representative of 3 independent experiments. Error bars (g) are SD and **p < 0.05 between saline and morphine treated groups for the same time-point]. (**f**) IL17 mRNA levels in 16HBE14o cells with LTA stimulation for 0.5, 1, 3, 6 and 24 hours and morphine modulation of the same. [Data representative of 3 independent experiments. Error bars are SD and #p < 0.05 between saline and morphine treated groups for the same time-point and **p < 0.05 compared to “no treatment placebo” group].

To understand the cellular source of the early-release IL-17A, we treated the WT animals with *Pneumococcal* lysate (PL; intra-nasally) for 3 hours as above and infused the lungs with Tissue-tek OCT medium (Sakura) via tracheal catheterization. The individual whole lungs were snap-frozen in liquid nitrogen and 5 μm sections were obtained using a cryostat (Leica). The sections were stained with FITC conjugated anti-IL-17A antibody (eBiosciences) with surprising outcome. Data shows the bronchial epithelial cells harboring a pool of IL-17A in the cytoplasm (Fig. 2A) in the untreated WT mice, with negligible difference in the pool upon 3 hours of PL administration (Supplementary Figure 1). Bronchial epithelial cell-line, 16HBE14o (kindly provided by Prof. Dieter Gruenert, UCSF) was used to understand the native response of pathogenic insult on the epithelial layer and effects of morphine on the response. Results shows that 16HBE14o cells do harbor constitutively expressed IL-17A cytokine within the cells (Fig. 2B). Since RORγt is one of the key transcription factors for the production of IL-17A, we set out to verify if the epithelial cells express this at the message and protein levels. RT-PCR data shows the presence of RORγt message in untreated cells, which does not change due to morphine treatment (Fig. 2C), western blot analysis shows a basal level presence of RORγt protein, further corroborating the constitutive presence of IL-17 in these cells (Fig. 2D; Supplementary Figure 2). The western blot further shows a spike in the protein levels at 3 hours and above-basal levels at 6 hours. The bronchial epithelial cell line, when stimulated with 2.5 μg/ml Lipotechoic Acid (LTA; TLR2 specific stimulation) exhibited a biphasic IL-17A response from the culture supernatant with peak response within as early as 30 minutes (Fig. 2E). Apart from the temporal differences, the maximal response looked similar to *in vivo* data in Fig. 1A with morphine mediated suppression of early-release IL-17A levels. Finally, with IL17 mRNA levels, we did not see any spike at 30 minutes of PL treatment, but a similar profile to the released cytokine level was observed beyond 6 hours of PL treatment (Fig. 2F), indicating that the early release of IL17 is independent of *de novo* synthesis of IL17, whereas 6 and 24 hours response is transcription dependent.

### Bronchial epithelial cells are the primary contributor to the TLR2 mediated early IL-17A release in the lungs which is independent of *de novo* synthesis

**Figure 3:**
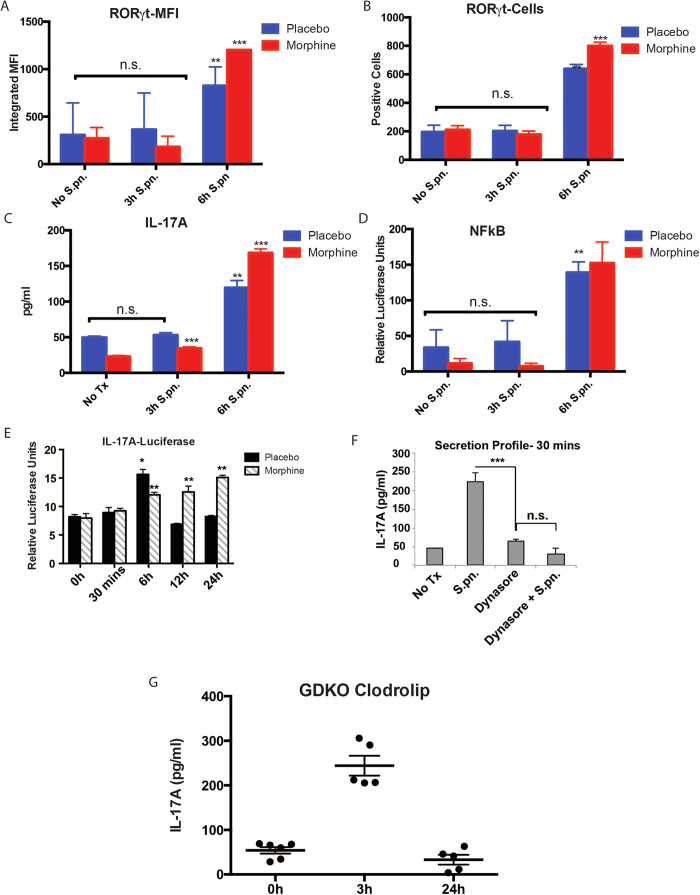
Bronchial epithelial cells are the primary contributor to the early TLR2 mediated IL-17 release in the lungs. BAL cells were harvested from C57Bl6/j animals (n = 5/condition), pre-conditioned with saline or 1 μM morphine for 24 hours and treated with 1 μg/ml PL for 0, 3 and 6 hours. Flow cytometry was performed to detect the RORγt positive cells for the indicated time points showing MFI (**a**) and absolute cell numbers (**b**) of RORγt positive cells. Culture supernatant from the same experiment was used to detect IL-17 levels (**c**) using sandwich ELISA [SD; n.s.=non-significant; **p < 0.05 compared to 0 or 3 hour time points for the respective treatment groups; ***p < 0.05 compared to placebo within the same time point]. (**d**) A separate set of animals (n = 4/condition) were used to obtain the BAL cells, which were pre-tranduced with a mix of lentivirus with NF-kB reporter (firefly luciferase) and control (renilla luciferase), pre-conditioned with saline or 1 μM morphine and treated with 1 μg/ml PL for 0, 3 and 6 hours. Luciferase activity was measured using Promega dual luciferase kit [SD; n.s.=non-significant; **p < 0.05 compared to 0 or 3 hour time points for the respective treatment groups]. (**e**) Human bronchial epithelial cells 16HBE14o were transfected with IL-17 reporter construct (firefly luciferase) and control (renilla luciferase), pre-conditioned with saline or 1 μM morphine and treated with 2.5 μg/ml LTA for 0, 0.5, 6, 12 and 24 hours. Luciferase activity was measured using Promega dual luciferase kit [SD; *p < 0.05 compared to 0 hour time point; **p < 0.05 compared to placebo for the respective treatment groups]. (**f**) 16HBE14o cells, pre-conditioned with 10 μM dynasore for 1 hour and treated with 1 μg/ml PL for 30 mins. IL-17 levels were measured using ELISA [SD; n.s.=non-significant; ***p < 0.05]. (**g**) ɣ∂ T cell deficient mice were given intra-nasal doses of clodrolip, thrice every 48 hours, before PL administration for 3 or 24 hours. “no PL” or 0 hours group was used as control. At specified time-points, animals (n = 4) were sacrificed and broncheo-alveolar lavage (BAL) was harvested using tracheal catheterization. IL-17 levels in the BAL was measured using ELISA [SD; n = 4; **p < 0.05 compared to 0 or 24 hour time points].

To verify the cell-type responsible for TLR2 mediated IL-17A release in the murine lung, BAL cells were harvested from WT animals and the mixed cell types were maintained on RPMI with or without morphine (1 μM) for 24 hours and treated with 1 μg/ml PL for 3 and 6 hours. The cells were collected, fixed and stained with FITC conjugated anti-RORγt antibody (eBiosciences) and analyzed with flow-cytometry. Median fluorescent intensity (MFI) of RORγt signal was calculated using Flowjo software (Treestar Inc.) and compared between the groups. Data shows (Fig. 3A,B) that between 0 and 3 hours, the RORγt levels do not show any changes either in saline or morphine pre-treated cells. After 6 hours of PL, we saw a modest, yet statistically significant up-regulation of RORγt in these cells. The culture supernatant from this experiment was used to measure IL-17A levels using ELISA (eBiosciences) and here too (Fig. 3C), we did not see any above basal release of IL-17A before 6 hours, corresponding with the RORγt profile. Similarly, a separate batch of BAL cells was pre-transduced with lentivirus, which would introduce NF-kB-binding consensus sequence driving luciferase expression. Cells were co-transduced with lentivirus with renilla luciferase under constitutive promoter as a transduction efficiency control. Cells were then pre-conditioned with placebo or 1 μM morphine for 24 hours and treated with 1 μg PL for 0, 3 or 6 hours and luciferase activity measured using the dual luciferase activity (promega; dual luciferase kit). Results show no NFkB activation at 3 hours with significant activity at 6 hours (Fig. 3D). Next, we set out to verify the IL-17A expression pattern in the 16HBE14o cells upon TLR2 stimulation. For that, the cell line was co-transfected with a PGL3 construct with firefly luciferase gene under the control of IL-17A promoter (Origene Inc.) and renilla luciferase under constitutive promoter. Transfected cells were treated with 2.5 μg/ml LTA and the firefly luciferase activity was measured at different time points and normalized with the renilla luciferase activity using the dual-luciferase kit (Promega). At 30 minutes post LTA treatment (where we see the peak IL-17A release from the cell line), there is no perceptible IL-17A expression activity (Fig. 3E), indicating that early release of IL-17A is independent of *de novo* synthesis. If we block exocytosis in these cells using 10μM Dynasore (inhibitor of dynamin 1,2, drp1 and mitochondrial dynamin)^27^,^28^,^29^, release of IL-17A upon TLR2 stimulation is seen to be prevented (Fig. 3F). Since a cohort of γδ T cells are known to constitutively express IL-17A and along with macrophages and dendritic cells, constitute the resident immune component in the lungs^30^,^31^, we treated γδ T cells knockout mice (GDKO) with clodrolip (clodronate coated vesicles which selectively block mitochondrial ATPase in phagosomes, hence inducing cellular apoptosis^32^,^33^) intra-nasally every 48 hours thrice. The animals were intra-nasally treated with PL, 24 hours after the third clodrolip treatment. BAL was harvested from these animals at indicated time-points and IL-17A levels measured (ELISA; eBiosciences). The IL-17A release pattern (Fig. 3G) was similar to the WT placebo animals as shown in Fig. 1. Efficacy of clodrolip was verified by measuring the depletion of F4/80+ macrophages from the BAL cells using flow-cytometry (Supplementary Figure 3).

### TLR2 mediated IL17 response exhibits tolerance in the lungs, which is modulated by chronic morphine

**Figure 4:**
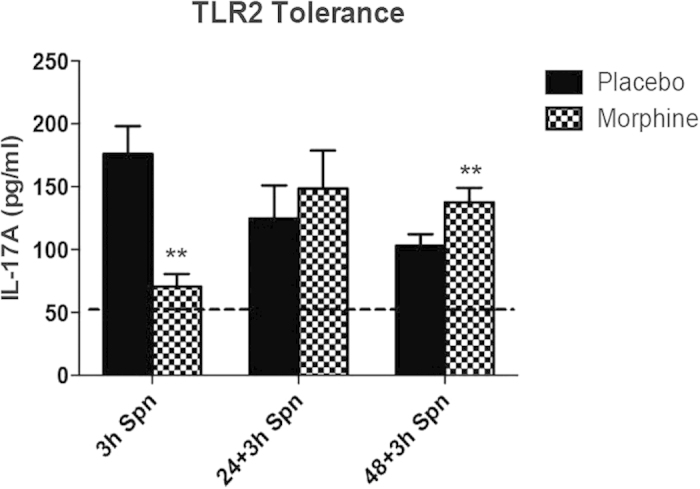
TLR2 mediated IL17 response exhibits tolerance in the lungs, which is modulated by chronic morphine. WT mice (C57BL/6) were implanted with placebo/morphine pellet for 24 hours and 100 μg PL intra-nasally administered to all animals. One cohort of animals was sacrificed after 3 hours of PL administration. After 24 hours, rest of the animals were given 100 μg PL again and the second batch of animals sacrificed after 3 hours of PL administration. The remaining animals were administered with 100μg PL after another 24 hours (48 hours after first PL administration) and sacrificed after 3 hours. Post euthanasia, BAL fluid was harvested for each animal via tracheal catherization, cell-free supernatant was obtained by centrifugation and IL-17 levels measured using ELISA (eBiosciences, San Diego, CA) [**p < 0.05 compared to the “placebo” control for the same time point; Horizontal line (---) represents basal level of IL17 in untreated WT mice].

Temporal differences in the secretion of TLR2 mediated IL17 from the epithelial cells (Fig. 2G) and downregulation of secretion after an initial surge, indicated towards TLR2 tolerance vis-à-vis IL17 secretion. To verify this *in vivo* using our tolerance model^3^, WT mice (C57BL/6) were implanted with placebo/morphine pellet for 24 hours and 100 μg PL intra-nasally administered to all animals. One cohort of animals was sacrificed after 3 hours of PL administration. After 24 hours, rest of the animals were given 100 μg PL again and the second batch of animals sacrificed after 3 hours of PL administration. The remaining animals were administered with 100 μg PL after another 24 hours (48 hours after first PL administration) and sacrificed after 3 hours. Post euthanasia, BAL fluid was harvested for each animal via tracheal catherization, cell-free supernatant was obtained by centrifugation and IL-17 levels measured using ELISA. Placebo implanted animals exhibit progressively decreasing IL17 levels in the BAL fluid for the indicated time points, while morphine implanted animals show an initial suppression and sustained levels of IL17 release at the later time points (Fig. 4).

### TLR2 and IL17R signaling intermediaries associate with each other and modulate the TLR2 dependent IL17 response

**Figure 5:**
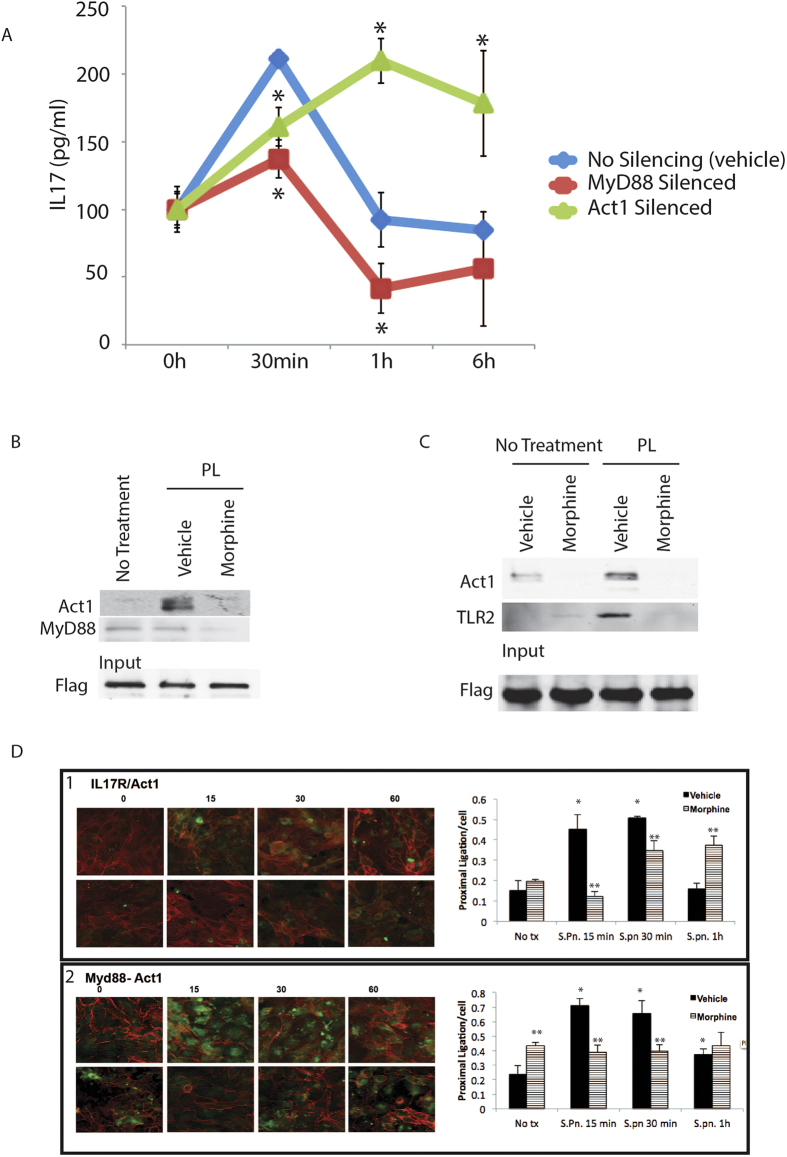
Time dependent association between the TLR2 and IL-17R adapter molecules. (**a**) siRNA pools and scrambled controls were used to silence the expression of MyD88 and Act1/CIKS in the Bronchial epithelial cell line, 16HBE14o. The cells were then stimulated with 1 μg/ml PL for 0, 0.5, 1 and 6 hours and IL-17 levels measured from the culture supernatant using ELISA [Error bars = SD; *p < 0.05 compared to the “no silencing” control for the same time point]. (**b**) 16HBE14o cells were transfected with flag-tagged IL-17R construct, pre-conditioned with saline or 1 μM morphine for 24 hours and stimulated with PL for 0.5 hours. Interactions were fixed by using DSS and IP was performed for flag and Act1 and MyD88 were detected by western blot. Pulled down flag was used as load control. Alternatively, (**c**) 16HBE14o cells were transfected with flag-tagged MyD88 construct, pre-conditioned with saline or 1 μM morphine for 24 hours and stimulated with PL for 0.5 hours. Interactions were fixed by using DSP and IP was performed for flag, Act1 and TLR2. (**d**) 16HBE14o cells were pre-conditioned with saline or 1 μM morphine for 24 hours and interaction of IL-17R-Act1 (panel 1) and MyD88-Act1 (panel 2), were measured using Proximal Ligation Assay (Please see methods) over time until an hour, post PL stimulation. Slides are counterstained with rhodamine-phalloidin (red) and punctate green color represents positive interaction between the antigen pair being studied. Histogram panels represent quantitation of PLA reaction using ImageJ [Error bar = SD; *p < 0.05 compared to “no tx” control; **p < 0.05 compared to placebo at the same time point].

Toll like receptors have been previously implicated in chronic lung inflammation^34^,^35^,^36^ and we^12^ and others^20^ have shown the role of TLR ligation leading to IL-17A production. We have verified that the bronchial epithelial cells do express the IL-17A receptor (IL-17R; Supplemenary Figure 4), which, in the context of lung opportunistic infection, would result in concurrent signaling through the two receptors with major overlap and redundancy in their signaling^37^. Hence, it would be logical to predict a cross talk between the two pathways at an early time point, as a mechanism to attain immune homeostasis after an initial robust response to infection. To verify this, we started with the first adaptor molecules of the TLR2 and IL17R pathways, namely MyD88 and Act1/CIKS respectively. MyD88 and Act1/CIKS expression was silenced (silencing verified with western blot in Supplementary Figure 5) in 16HBE14o cells and IL-17A levels were measured from the culture supernatant using ELISA. Results (Fig. 5A) show a robust IL17 response at 30 minutes for the vehicle control, which wanes down by one hour and maintains at basal-level till 6 hours. Silencing MyD88 significantly obliterates TLR2 mediated IL17 response at all time points indicating a signaling shut-down, whereas Act1/CIKS silencing shows significant build-up of IL17 at all treatment time points. This indicates that presence of Act1/CIKS has an important part in TLR2 silencing and silencing this adaptor mimics morphine effect in IL-17A secretion. Next, we wanted to verify if the first adaptor molecules associate with each other in real-time within the cell. For that, 16HBE14o cells were transfected either with a flag-tagged IL-17R or MyD88 constructs (both Origene), preconditioned with saline/1 μM morphine for 24 hours and treated with 1 μg/ml PL for 30 minutes. Protein interactions were fixed using a homobifunctional cross linker Disuccinimidyl Suberate (DSS; Pierce) and the cells lysed and a pull-down assay was performed using anti-flag antibody (sigma) and the interacting complex eluted with 3X flag peptide (Sigma). Western blot results (Fig. 5B; Supplementary Figure 6) with IL-17R-flag pull down shows that MyD88 and Act1 associate with their respective receptors and with each other within 30 minutes of TLR2 stimulation, which is disrupted by morphine. Additionally, pull down with myD88-flag (Fig. 5C; Supplementary Figure 7) shows that MyD88 and Act1 pre-exist as a complex within the cell. Based on these results, we hypothesize that MyD88 and Act1 associate with each other in a putative pre-formed complex within the cells and morphine disrupts this complex, thereby rendering the adaptors to be available for the respective receptors at later time points, leading to uncontrolled pro-inflammatory signaling. To focus further on the Act1-myD88 interaction, based on a well-established paradigm of IL-17R-Act1 interaction^38^,^39^, we performed the proximal ligation assay (PLA; Olink Biosciences; www.olink.com), based on *in situ* ligation and amplification of probe oligonucleotides, depending on proximity of interaction between two molecules^40^. Based on the interaction characteristics shown before^38^,^39^,^41^, we verified the interaction dynamics between IL-17R and Act1/CIKS (Fig. 5D panel 1), where we show increased interaction between the two molecules upon IL-17A ligation till 30 minutes and return to basal level interaction within 1 hour. Morphine treated samples, show slower interaction kinetics for the same time points. Next, we looked at the interaction between Myd88 and Act1/CIKS under the same conditions and found a similar association dynamics as Il-17A-Act1/CIKS (Fig. 5D panel 2). Morphine however, did not show any interaction across any time point. This verified further that the first messenger molecules for the two pathways have unique association related to TLR2 mediated early IL-17A release.

### TLR2 mediated early IL-17A release modulates lung barrier integrity

**Figure 6:**
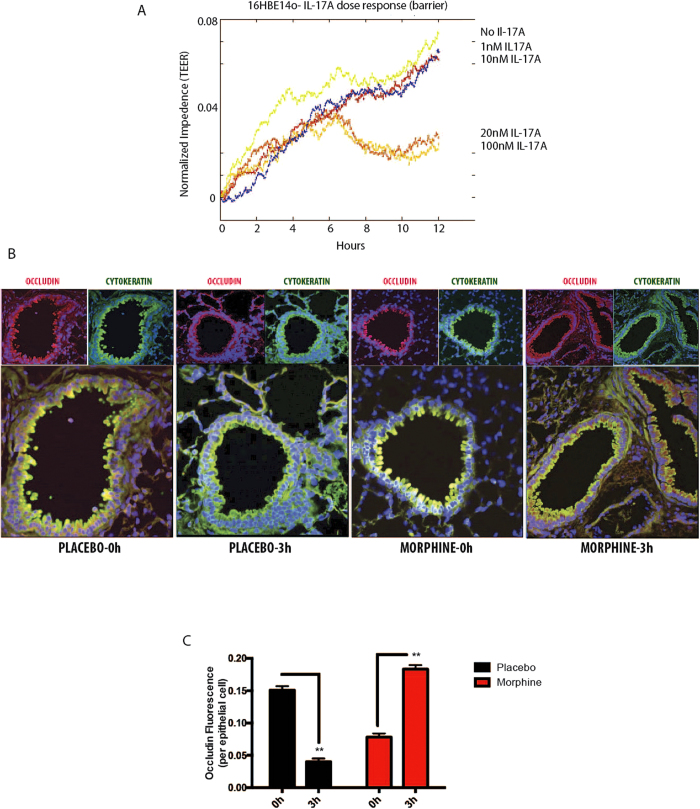
IL-17 and its differential effects on epithelial barrier integrity. (**a**) 16HBE14o cell line was plated on electrode array chamber slides and allowed to be confluent. Upon confluence, they were treated with 0, 1, 10, 20 and 100 nM recombinant IL-17 and trans-epithelial electrical resistance (TEER; impedence) was measured in real-time to verify the effects of the cytokine on epithelial barrier. There is a clear departure of bronchial epithelial response to change in IL17 concentration; while lower doses (up to 10 nM) are protective, higher doses (20 and 100 nM) exhibit disruption in barrier integrity. (**b**) Wild-type (C57Bl6/j) animals (n = 5) were implanted with placebo/75 mg morphine pellets for 24 hours and PL was intrasasally administered for 0 and 3 hours. Lung sections from these animals were stained for occludin (tight-junction protein) and cytokeratin (epithelial marker) to verify the status of barrier. Fluorescence intensity of occludin was measured using ImageJ (**c**) and normalized with cytokeratin MFI [error bar = SD; **p < 0.01].

IL-17A has been variously implicated in both protective^17^ and disruptive^42^ effects on mucosal integrity. The contemporary understanding emerging from the literature indicates that the dual functionality associated with this cytokine might be dose dependent. To test this, we used the 16HBE14o cells and subjected it to Electric Cell-substrate Impedance sensing (ECIS; Applied Biophysics) with escalating doses of recombinant IL-17A (rIL-17A; ebiosciences). The cells were allowed to reach confluence, rIL-17A added and impedance was measured for 12 hours (Fig. 6A). Result shows a clear dose-dependent departure in barrier formation/integrity, where no IL-17A, 1 nM and 10 nM dose allowed the establishment of epithelial barrier; 20 nM and 100 nM disrupted the formation of an intact barrier. Despite the difficulty in correlating the *in vitro* barrier information with the *in vivo* results thus far, we set out to determine the status of epithelial barrier in WT mice at 3 hours post TLR2 stimulation (peak IL-17A response; Fig. 1A). WT mice were implanted with placebo or morphine pellets as before (24 hours) with or without intranasal PL administration for 3 hours. Animals were sacrificed and 10% neutral formalin was infused into the lungs by tracheal catheterization and processed for paraffin embedding. Approximately 5μm sections were generated (Pathology; University of Minnesota), de-paraffinized and stained for pan-cytokeratin (green; epithelial marker) and occludin (red; marker for epithelial tight junction). Representative immunofluorescence micrograph (Fig. 6B) and Median Fluorescence Intensity (MFI) calculations from 5 animals per group (Fig. 6C) show a subtle disassociation of occludin from the luminal epithelia 3 hours post PL administration. Morphine by itself, does not seem to have a major effect on the barrier status at this time point. PL administration with chronic morphine, however, seems to robustly reinforce occludin mobilization on the epithelial surface. While it is easy to hypothesize that PL administration and resultant IL-17A release results in transient compromise in barrier, with reverse effect when morphine is on board, the physiological significance of this phenomenon was not very clear at this point.

### TLR2 mediated early IL-17A release is required for early neutrophil recruitment

**Figure 7:**
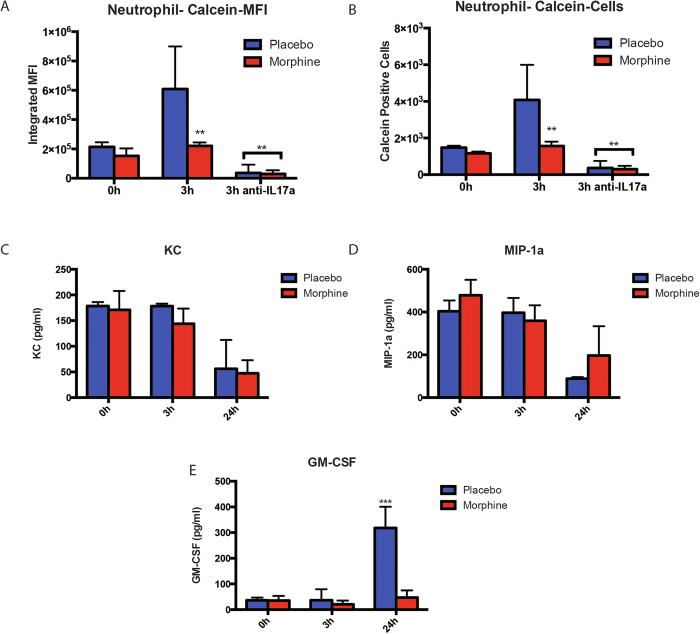
TLR2 mediated IL-17 release and neutrophil recruitment. (**a**) Peripheral neutrophils were isolated and purified from a cohort of WT/C57Bl6/j animals, labled with calceinAM and injected *i.v.* into 3 groups of WT animals (n = 4), pre-implanted with placebo or 75 mg slow-release morphine pellets for 24 hours. Allowing 24 more hours for even spread of labeled neutrophils in the mice, intra-nasal administration of saline, 100 μg PL and 100 μg PL + anti-IL-17 blocking antibody was done in separate groups of animals for 3 hours. MFI of green fluorescence (calcein; freshly recruited neutrophils) among BAL cells were measured with flow-cytometry and analyzed with FloJo software [*p < 0.01 compared to all other groups; **p < 0.05 compared to 3 h placebo]. (**b**) Depicts number of calcein-positive cells from the experiment described in (a) [*p < 0.01 compared to all other groups; **p < 0.05 compared to 3 h placebo]. In a separate experiment, 3 groups of WT mice were implanted with placebo or 75 mg slow-release morphine pellets for 24 hours and 100 μg PL was intra-nasally administered to each animal. Groups of animals (n = 4) were sacrificed at 0, 3 and 24 hours and (**c**) KC, (**d**) MIP-1a and (**e**) GM-CSF levels were measured from the BAL fluid using ELISA [***p < 0.01 compared to all other groups].

The role of IL-17A and IL-17R signaling in MIP-2 and KC mediated neutrophil recruitment is well documented by others and us^10^,^12^,^19^,^43^,^44^,^45^. At the same time, there has been evidence based studies, where the possibility if IL-17A being able to recruit neutrophil to the site of inflammation by itself or with the help of a concurrent IL-8/KC release has been postulated. To verify whether neutrophil recruitment was one of the functions served by pathogen mediated IL-17A release, we isolated and purified neutrophils from the peripheral murine blood and metabolically labeled them with Calcein AM (molecular probes) and injected them (*i.v.*) into three groups of animals along with implanting them with placebo/morphine as appropriate. The animals were intranasally administered with PL or PL spiked with anti-IL-17A blocking antibody for 3 hours and BAL harvested. The BAL cells were analyzed with flow-cytometry and MFI of recruited green cells were analyzed (Fig. 7A,B). Data shows infiltration of neutrophils into the lung lavage upon TLR2 stimulation and IL-17A release, which is completely obliterated in animals receiving IL-17A blocking antibody. For the same time points, we also measured the already implicated chemokines responsible for neutrophil recruitment^12^, namely MIP-2, KC and GMCSF and did not see any up-regulation at 3 hours (Fig. 7C–E respectively), indicating a direct role of IL-17A in modulating the barrier integrity of lung mucosa and neutrophil recruitment at a very early stages of infection. GM-CSF showed a significant up-regulation at 24 hours (Fig. 7E), indicating its role in neutrophil recruitment at a later stage. At the same time, chronic morphine subdues this early response, thereby disrupting a tightly controlled resolution cascade at a very early stage.

## Discussion

Lung infection/inflammation occupies a pivotal position in the world health organization (WHO) disability-adjusted life years (DALY) lost, followed by HIV/AIDS and neoplasms (benign or malignant)^4^. Respiratory infection has been recognized as the prime cause of mortality in the clinics for adult acute leukemia^46^, and extreme risk factor for acquired pneumonia in hematological malignancies^47^. In most cases, clinical care involves the usage of opioids for pain management, specifically aligned with the treatment of various cancers and AIDS^3^,^4^,^5^. Drug abuse, combined with the aforementioned morbidities present an added complexity in clinical care of a significant cohort of patients by making them susceptible to secondary infections, predominantly to *pneumococcus*^6^,^10^,^14^,^15^. We have shown before that one of the primary cytokine responses to *pneumococcus* infection in lungs is mediated through a biphasic IL-17 release^12^ starting at 4 hours. This study, as an extension, shows that the primary IL-17 response peaking as early as 3 hours post infection in our murine model and that the initial response is blunted in morphine implanted mice (Fig. 1A). We verified that this initial blunting is μ-opioid receptor dependent by looking at the receptor knockout (MORKO) animals, where this effect was completely abrogated at 3 hours (Fig. 1B). While TLR2 is known to stimulate IL-17 response^18^,^20^, *pneumococcus* infection is also known to stimulate TLR4 to a lesser extent^48^, which prompted us to check this phenomenon in the respective receptor knockout animals. While TLR4KO animals showed a similar IL-17 profile as the wild-type, it was severely muted in TLR2KO animals at 3 hours for both placebo and morphine implanted animals (Fig. 1C,D). Hence, it was clear that the initial IL-17 release is TLR2 and not TLR4 dependent. We also observed attenuated response at 24 hours response in TLR4KO animals. There is enough evidence in the literature and our own work^10^,^12^ that IL17 release follows a biphasic response pattern. Here, we have shown that the early response (3 hours) is TLR2 dependent. The second response (24 hours) seems to be both TLR2 and 4 dependent. Indeed, if we neutralize MyD88 (inhibitor peptide) in the Human bronchial epithelial cells, *Pneumococcus* lysate significantly attenuated IL17 response at 24 hours (Supplementary Figure 8), similar to what we see in Fig. 5A in the manuscript (up to 6 hours).

One of our initial concerns with the time point (3 hours) of the IL-17 response was that this cytokine release pre-empted any likelihood of cellular *de novo* synthesis following TLR2 signaling. Our initial hypothesis was infiltration of IL-17 producing cells due to chemokine release by lung tissues as insinuated before^20^. To verify this we implanted wild-type, TLR4KO and TLR2KO mice with placebo or morphine pellet for 24 hours and 3 hours post intranasal PL administration, animals were sacrificed, lungs infused with OCT medium, sectioned in cryostat and stained for IL-17. Data showed that the principal cell-type with strong IL-17 staining was the bronchial epithelial cells for the wild-type (Fig. 2A), with non-detectable change 3 hours post PL administration (Supplementary Figure 1). While the lack of difference in staining between the two time points could be attributed to the modest initial response at 3 hours and non-quantitative nature of the method, it was amply clear that the bronchial epithelia harbored a pool of IL-17, which was presumably released upon TLR2 stimulation.

While this data was unexpected, upon literature survey, we realized that in several publications depicting IL-17 staining in murine or human lungs, have shown mild to moderate stain on the bronchial epithelia and attributed it to non-specific staining^19^. Additionally, in the past decade, the role of bronchial epithelia has been expanded as one of the essential cell-type in the induction of pathogenic resistance in its own right^21^,^22^,^23^. To explore this aspect, we used the human bronchial epithelial cell line 16HBE14o, which maintains polarity under specific culture conditions^49^. Under the prescribed culture conditions (see methods), these cells stain positive for intracellular IL-17 (Fig. 2B) and maintain a steady-state message and protein level of the key IL-17 regulating transcription factor RORγt (Fig. 2C,D) and exhibit a biphasic IL-17 release in response to TLR2 stimulation (Fig. 2E) with morphine mediated inhibition of the early release of the cytokine. It is worth noting here that the initial IL-17 response *in vitro* is as early as 30 minutes, compared to 3 hours in *in vivo* conditions. We believe this is due to differential accessibility of the antigen to the receptors under the two experimental conditions. For IL17 mRNA levels, we did not see any spike at 30 minutes of PL treatment, but a similar profile to the released cytokine level was observed beyond 6 hours of PL treatment (Fig. 2F), indicating that the early release of IL17 is independent of *de novo* synthesis of IL17, whereas 6 and 24 hours response is transcription dependent.

Based on our data so far and indications on the expanded role of bronchial epithelia^22^, we tried to understand the primary contributor cell-type for the early-release IL-17 in the lungs using *in vitro* PL stimulation of total BAL cells from WT animals. We did not see any RORγt up-regulation (Fig. 3A), IL-17 release (Fig. 3B) or NF-kB activation (Fig. 3C) in the first 3 hours post-stimulation. For these parameters, the first incidence of moderate to significant changes happened 6 hours post stimulation, corresponding to the second phase of IL-17 release, suggesting that the early-release IL-17 was not derived from the BAL cells and independent of *de novo* synthesis. Similar trend was observed in the 16HBE14o cell-line, where we did not see any IL-17 promoter activity corresponding with the early IL-17 release (Fig. 3D). Additionally, the early IL-17 response was abrogated by inhibiting the secretion apparatus (Fig. 3E). These experiments, combined with a comparable IL-17 response in GDKO animals, depleted of macrophages and other phagocytes (Fig. 3F), indicated that the bronchial epithelium is the major contributor to the early-release IL-17 in response to TLR2 stimulation.

The overlap and redundancy between the TLR2 and IL-17R signaling^37^ presents a unique problem apropos morphine mediated early suppression and over secretion of IL-17 at a later time point. Based on our established tolerance model^3^, we looked at TLR2 silencing based on IL-17 release and clearly saw morphine mediated abrogation of tolerance in the lungs (Fig. 4). We hypothesized that cross-talk between the two pathways might be affecting tolerance, which is being tempered by morphine. Our data shows that while silencing the first adapter molecule of TLR2 pathway (MyD88) prevents IL-17 release, silencing the first adapter of IL-17R pathway (Act1) mimics the morphine effect, namely early suppression and above-basal IL-17 levels at later time point (Fig. 5A). Pull-down assays with IL-17R and MyD88 (Fig. 5B,C) demonstrated that morphine was not suppressing the expression of Act1, but dissociating interaction between MyD88 and Act1, indicating that TLR2 tolerance with respect to IL-17 release is indeed brought about by interaction between the two molecules, thereby making them “unavailable” to their respective receptors, effecting pathway shutdown. Morphine seems to affect a dissociation of this complex, making the adapters “available” to the receptors resulting in abrogation of tolerance. Using PLA, we further show that morphine indeed results in dissociation of MyD88-Act1, otherwise interacting with each other upon TLR2 stimulation (Fig. 5D).

The whole mechanism of TLR reprogramming/tolerance is understood to play a very important role in restoring immune homeostasis following a cytokine surge due to pathogen attack^3^,^50^. Morphine, in the context of TLR2 reprogramming, in this instance, modulates IL-17 release, which could have differential effects on the key steps involved in the resolution of inflammation^12^, namely mucosal barrier integrity and neutrophil recruitment. The dual effect of IL-17 is evident from the literature^17^,^42^ and we have shown a dose-dependent protection/disruption of bronchial epithelium (Fig. 6A). In WT mice, we show that 3 hours post TLR2 stimulation, higher IL-17 levels result in transient loosening of barrier (Fig. 6B,C), likely to facilitate infiltration of neutrophils (Fig. 7A,B), whereas morphine suppression of IL-17, results in intact/reinforced barrier, inhibiting neutrophil recruitment. The phenomenon of early neutrophil recruitment too, seems to be IL-17 dependent (Fig. 7A,B). We have verified that the previously implicated chemokines, namely MIP-2, KC and GM-CSF do not play a direct role in this phase of neutrophil recruitment (Fig. 7C–E), but its sole dependence on IL-17 could not be verified.

**Figure 8:**
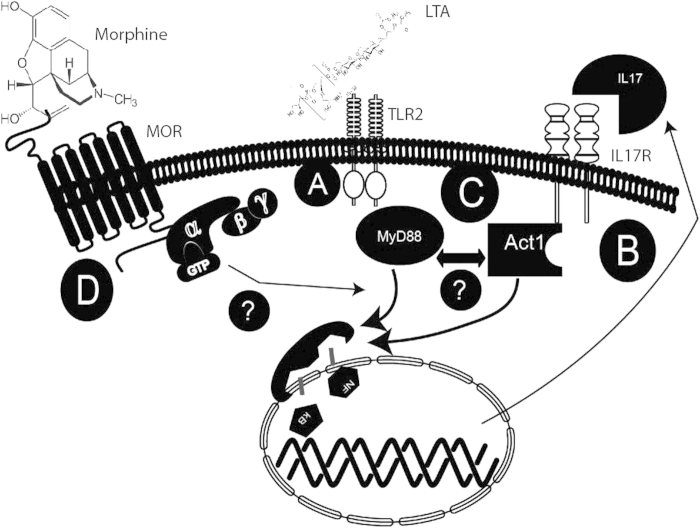
Proposed model for IL-17 homeostasis in the lungs and its disruption by morphine. IL17 levels are elevated in the broncho-alveolar lavage (BAL) of wild-type mice following infection with *S. pneumoniae*. Additionally, we have shown that bronchial epithelial cells have the requisite machinery to produce and secrete IL-17, upon TLR2 stimulation. We propose that TLR2 activation resulting in IL-17 secretion and build-up in the lung (**a**) stimulates IL17R which signals through Act1 and TRAF6 to downregulate TLR2 induced pro-inflammatory cytokines including IL-17 after an initial burst (**b**). Our data shows that 1) epithelial TLR2 mediated IL-17 secretion is the first line of defense against invading pathogens. 2) Cross talk between IL-17 induced IL17 receptor activation and TLR signaling results in down-regulation of pro-inflammatory factors (TLR2 tolerance) after the initial burst and returns homeostatic balance, implying a protective inhibitory loop in the inflammatory milieu of the two pathways (**c**) and, 3) Morphine treatment disrupts the cross talk thus upsetting the homeostatic balance. Our preliminary studies indeed suggest association between downstream signaling intermediaries between the two pathways supporting chronic morphine’s disruption of this cross-talk (**d**), upsetting the tight balance that keeps the inflammation in check under normal conditions.

In this study, we show that early induction of IL17 following pathogenic encounter is a protective response against pathogens in the lungs, which has so far been attributed to TLR2 activation in the circulating immune cells. We also show that the bronchial epithelial cells have the requisite machinery to produce and secrete IL17A, upon TLR2 stimulation. TLR2 activation resulting in IL17A secretion and build-up in the lung (Fig. 8A) stimulates IL17R and triggers NF-κB mediated pro-inflammatory cytokine/chemokine cascade (Fig. 8B), similar to TLR2 signaling. We hypothesize that 1) epithelial TLR2 mediated Il-17A secretion is the first line of defense against invading pathogens. 2) Cross talk between IL17A induced IL17 receptor activation and TLR signaling results in down-regulation of pro-inflammatory factors (TLR2 tolerance) after the initial burst and returns homeostatic balance, implying a protective inhibitory loop in the inflammatory milieu of the two pathways (Fig. 8C) and, 3) Morphine treatment disrupts the cross talk thus upsetting the homeostatic balance. Our data indeed suggest association between downstream signaling intermediaries between the two pathways supporting chronic morphine’s disruption of this cross-talk (Fig. 8D), upsetting the tight balance that keeps the inflammation in check under normal conditions. This study provides clinically relevant information in terms of therapeutics, by identifying a static target (bronchial epithelia) and defined pathways and receptors (TLR2 and IL17R), compared to a systemic intervention (circulating immune cells) in the context of respiratory inflammation and morphine.

## Methods

All methods were carried out in accordance with the approved guidelines and policies.

### Mice and Cell Lines

C57BL/6 mice were purchased from Jackson Laboratories (Bar Harbor, Maine). TLR2KO, TLR4KO and MORKO mice were maintained in-house. All animals were maintained in pathogen-free facilities and all procedures were approved by the University of Minnesota Institutional Animal Care and Use Committee. Typically, 8–10 week old male animals were used for our studies. Human bronchial epithelial cell line, 16HBE14o was kindly provided by Prof Dieter Gruenert (UCSF).

### Pneumococcus Lysate (PL) preparation

*Streptococcus pneumoniae* (T3) culture was grown to exponential phase in BHI medium (BD, Franklin Lakes, NJ) and the bacterial cells harvested with centrifugation. The cells were resuspended in minimum volume of PBS and lysed by ultrasonic pulses till the solution became clear. Total protein from the post-debris supernatant was quantitated using BCA assay kit (Pierce, Rockford, IL) and the volume adjusted to 2μg/μl with PBS, aliquoted and saved at −80° until required.

### Placebo/Morphine pellet Implantation

Slow release morphine pellets (75 mg; 1.2 μM steady serum levels for up to 10 days) and corresponding placebo pellets were kindly provided by National Institute of Drug Abuse (NIDA, National Institutes of Health, Rockville, MD). The implantation procedure involved 3% isoflurane induced anesthesia, followed by making a small incision at the dorsal torso of the mice. The appropriate pellet was inserted into the small pocket created during incision and the wound was closed using stainless steel wound-clips. The whole process was carried out under aseptic conditions.

### Cell Culture and Treatments

16HBE14o cells were cultured at 37 °C with 5% CO_2_ in 2% fibronectin coated culture-ware in Bronchialife supplemented media (Lifeline Cell Technology, Frederick, MD). For transfection of different plasmids (all constructs from Genecopoea, Rockville, MD) X-tremegene HP transfection reagent (Roche, Indianapolis, IN) was used according to the manufacturer’s specifications. For *in vitro* IL-17 measurements, the cells were preconditioned with 1 μM morphine sulphate (Sigma, St. Louis, MO) or PBS for 24 hours and treated with 2.5 μg/ml LTA (Sigma, St. Louis, MO) for 0, 0.5, 1, 2, 3 and 6 hours. At each time point, the culture supernatant was saved and ELISA was performed using IL-17 easy-set-go ELISA kit (ebiosciences, San Diego, CA) as per manufacturer’s instructions. For *in vitro* luciferase assays, 16HBE14o cells were transfected with IL-17 reporter construct (firefly luciferase) and control (renilla luciferase), pre-conditioned with saline or 1 μM morphine and treated with 2.5 μg/ml LTA for 0, 0.5, 6, 12 and 24 hours. Luciferase activity, as a surrogate for IL-17 synthesis, was performed using dual luciferase kit (Promega, Madison, WI).

### Dynasore Treatment

Human bronchial epithelial cell line, 16HBE14o was grown on fibronectin-coated 12-well plates and allowed to reach 75–80% confluence. The cells were then pre-conditioned with 10 μM dynasore for 1 hour and treated with 1 μg/ml PL for 30 mins. IL-17 levels were measured from the culture supernatant using ELISA (eBiosciences, San Diego, CA).

### Murine BAL cell isolation and *ex-vivo ana*lysis

C57BL/6 mice were implanted with placebo or 75 mg slow-release morphine pellet for 24 hours. BAL cells were harvested by tracheal catherization, followed by washing and harvest using 2-3 ml PBS. Mixed cell types were maintained on RPMI with or without morphine (1 μM) for 24 hours and treated with 1 μg/ml PL for 3 and 6 hours. Median fluorescent intensity (MFI) of RORγt signal was calculated using Flowjo software (Treestar Inc, Ashland, OR) and compared between the groups. Alternatively, a separate batch of animals were pre-transduced with lentivirus, which would introduce NF-kB-binding consensus sequence driving luciferase reporter expression (Qiagen, Germantown, MD). Animals were co-transduced with lentivirus with renilla luciferase under constitutive promoter as a transduction efficiency control, followed by Placebo/morphine pellet implantation. Animals were intra-nasally treated with 100 μg PL for 0, 3 or 6 hours and BAL cells were harvested as mentioned above. Dual luciferase activity was measured using the dual luciferase assay kit (Promega, Madison, WI) as mentioned above.

### *In vivo* Lentivirus administration

For *in vivo* administration of lentiviral particles, approximately 10^7^ TU/mice were intra-nasally administered, at least twice every 48 hours, followed by placebo/morphine pellet implantation. PL was intra-nasally administered 24 hours post pellet implantation as appropriate.

### *In vivo* clodrolip administration

Clodronate (Dichloromethylene Biphosphonic acid), encapsulated in liposomes was purchased from Encapsula nanosciences (Brentwood, TN) along with control empty liposomes. γδ T cells deficient mice (GDKO) were intra-nasally administered with 40 μl of the commercial formulation every 48 hours three times. PL was administered intra-nasally 48 hours after the final clodrolip administration for 3 and 24 hours. The 0 hour PL control animals got the control liposomes, similar to the clodrolip regimen mentioned above.

### *In vivo* tolerance studies

WT mice (C57BL/6) were implanted with placebo/morphine pellet for 24 hours and 100 μg PL intra-nasally administered to all animals. One cohort of animals were sacrificed after 3 hours of PL administration. After 24 hours, rest of the animals were given 100 μg PL again and the second batch of animals sacrificed after 3 hours of PL administration. The remaining animals were administered with 100 μg PL after another 24 hours (48 hours after first PL administration) and sacrificed after 3 hours. Post euthanasia, BAL fluid was harvested for each animal via tracheal catherization, cell-free supernatant was obtained by centrifugation and IL-17 levels measured using ELISA (eBiosciences, San Diego, CA).

### *In vitro* silencing

Human bronchial epithelial cell line, 16HBE14o was grown on fibronectin-coated 6 well plates (Corning, NY) to 60–65% confluence and transfected with FlexiTube siRNA premix (Qiagen, Germantown, MD) against MyD88 and Act1/CIKS. Scrambled siRNA was used as a control. The cells were transfected twice within 48 hours. Cells from replicate wells were used for verification of silencing with western blot and the experimental wells were stimulated with 1 μg/ml PL for 0, 0.5, 1 and 6 hours and IL-17 levels measured from the culture supernatant using ELISA (eBiosciences, San Diego, CA).

### Trans-Epithelial Impedance Measurements

Human bronchial epithelial cell line, 16HBE14o was grown on fibronectin-coated 8W10E+ electrode arrays (Applied Biophysics, Troy, NY) and allowed to reach confluence. Upon confluence, they were treated with 0, 1, 10, 20 and 100 nM recombinant IL-17 (eBiosciences, San Diego, CA) and trans-epithelial electrical resistance (TEER; impedence) was measured in real-time using ECIS-ZΘ (Applied Biophysics, Troy, NY) for 12 hours. Data was obtained as an average of 4 replicates per condition.

### Cytokine/Chemokine assays

IL-17/KC/MIP-1a/GM-CSF ELISA kits were purchased from eBiosciences (San Diego, CA) and assay performed according to the manufacturer’s protocol. For each assay, culture supernatant or BAL fluid was quantitated with BCA protein assay kit (Pierce, Rockford, IL) and cytokine/chemokine levels were determined for 1 mg total protein load.

### PCR and Primers

For PCR studies, 16HBE14o cells were pre-conditioned with saline/morphine as above and the cells were harvested at 3 and 24 hours of LTA treatment in 1ml Trizol reagent (Invitrogen, Grand Island, NY) and processed for RNA isolation, or stored in −80° freezer until further processing. Cellular RNA was extracted using TRIzol (Invitrogen, Grand Island, NY), and cDNA was synthesized with the M-MLV Reverse Transcription Kit (Promega, Madison, WI). Primers for RORγt and 18S ribosomal RNA were purchased from IDT (Coralville, IA). Polymerase chain reaction (PCR) was performed on an Applied Biosystems 7500 Realtime PCR Detection system. All samples were run in triplicate, and relative mRNA expression levels were visualized on a 1% agarose gel. Primer sequence:18s 5′-GTAACCCGTTGAACCCCATT-3′; 5′-CCATCCAATCGGTAGTAGCG-3′ RORγt: 5′-GAGGAAGTCCATGTGGGAGA-3′; 5′-TCCTAACCAGCACCACTTCC-3′; huIL17A 5′-CATGAACTCTGTCCCCATCC-3′; 5′-CCCACGGACACCAGTATCTT-3′ All samples were run in triplicate, and relative mRNA expression levels were analyzed using 18s as loading control.

### Western Blot and Pull-Down Assays

All antibodies for western blot analysis were purchased from Cell Signaling Technologies (Danvers, MA). Pull-down antibodies were purchased from Sigma (St. Louis, MO). Electrophoresis was performed on AnyKd mini gels (Bio-Rad, Hercules, CA) and transferred onto nitrocellulose membranes (Bio-Rad, Hercules, CA). The membranes were typically blocked with G-blocker (G Biosciences, St. Louis, MO) overnight and probed with primary antibodies for 2 hours. Membrane was incubated with IRdye (680 and 800; Licor, Lincoln, NE) for one hour, which allowed us to detect the loading control in the same membrane. Signal acquisition was done on Odyssey western blot developer (Licor, Lincoln, NE) as per manufacturer’s instructions. For pull-down assays, 16HBE14o cells were cultured to confluence and transfected with flag-tagged IL-17R or MyD88 expression construct (Genecopoea, Rockville, MD). The cells were pre-conditioned with saline/1 μM morphine 48 hours post transfection for another 24 hours and treated with PL for 30 mins. At the end of 30 mins incubation, the molecular interactions were fixed using thiol-cleavable homo-bifunctional cross-linker Disuccinimidyl Propionate (DSP; Pierce, Rockford, IL) for 5 minutes, reaction neutralized with 50 mM Tris.Hcl and cells lysed with RIPA buffer (Pierce, Rockford, IL). The cell lysate was incubated with the flag capture resin (Sigma, St. Louis, MO) for 2 hours, washed with PBST and the bound fraction eluted with 3X flag peptide (Sigma, St. Louis, MO). The eluted fractions were boiled with SDS-PAGE sample buffer (Pierce, Rockford, IL), which also reverses the DSP cross-linking. Samples were run on SDS-PAGE and western blot was performed as mentioned earlier.

### Proximal Ligation Assay

Human bronchial epithelial cell line, 16HBE14o was grown on fibronectin-coated 8 chamber slides to confluence and pre-conditioned with saline/1 μM morphine for 24 hours. One set of chamber slides were treated with 20 nM recombinant IL-17 in a reverse temporal fashion and reaction stopped at the same time to have IL-17 exposure at 15, 30 and 60 minutes for different replicate wells. The slides were fixed with 10% formalin (Sigma, St Louis, MO), washed twice and permeabilized using permeabilizing buffer (eBiosciences, San Diego, CA). A separate set of chamber slides was processed as before using 1 μg/ml PL instead of rIL-17. Post permeabilization, the cells were incubated for 2 hours with a set of IL-17R-Act1 or MyD88-Act1 primary antibodies (all antibodies from Sigma, St Louis, MO) and appropriate PLA probes (secondary antibodies with complementary oligonucleotides, Olink Inc., Uppsala, Sweden) with intervening washing steps and proceeded with the PLA assay as per manufacturer’s instructions. Upon completion, the slides were counterstained with rhodamine-phalloidin (Sigma, St Louis, MO) and images obtained with a Leica DM5500 immunofluorescence microscope. Image analysis and quantitation was done with ImageJ software (NIH). In this experiment, green fluorescence was used as the reporter and appearance of green fluorescence depicts less than 10-angstrom distance between the two antigens being tested; the minimum distance required for molecular interactions.

### Staining and Microscopy

Wild-type (C57Bl6/j) animals (n = 5) were implanted with placebo/75 mg morphine pellets for 24 hours and PL was intrasasally administered for 0 and 3 hours. Lung sections from these animals were stained for occludin-APC (tight-junction protein) and cytokeratin-FITC (epithelial marker) to verify the status of barrier (both antibodies from eBiosciences, San Diego, CA). Images were obtained with a Leica DM5500 immunofluorescence microscope and fluorescence intensity of occludin was measured and normalized with cytokeratin MFI using ImageJ software (NIH).

### Adaptive transfer and metabolic labeling

Neutrophils were isolated and purified from the peripheral blood of a cohort of C57Bl6/j mice using the neutrophil isolation kit (Miltenyi Biotech, San Diego, CA). Purified neutrophils were metabolically labeled with Calcein AM (molecular probes, Grand Islands, NY) and injected (*i.v.*) into three groups of animals. Animals were implanted with placebo/morphine pellets as appropriate. Up to 24 hours were allowed for the labeled neutrophils to spread and attain systemic homeostasis. The animals were intranasally administered with PL or PL spiked with anti-IL17 blocking antibody for 3 hours and BAL harvested and recruitment of labeled cells were analyzed using flow cytometry.

### Statistical Analysis

Cytokine concentrations from culture supernatant were expressed as change versus saline or “no treatment” control ±SD. For BAL level changes, this was expressed as ±SD. Fold changes in qPCR were expressed as ±SD between groups. Significance was defined as p < 0.05 (ELISA) and p < 0.01 (qPCR) in an unpaired student’s t test. For multivariate analysis, a two-way ANOVA was used. All statistical analysis was done with Prism (GraphPad, La Jolla, CA).

## Additional Information

**How to cite this article**: Banerjee, S. *et al.* Morphine compromises bronchial epithelial TLR2/IL17R signaling crosstalk, necessary for lung IL17 homeostasis. *Sci. Rep.* **5**, 11384; doi: 10.1038/srep11384 (2015).

## Supplementary Material

Supplementary Information
