# Supplementary material for: Morphine compromises bronchial epithelial TLR2/IL17R signaling crosstalk, necessary for lung IL17 homeostasis
Source: Sci Rep. 2015 Jun 15;5:11384. doi: 10.1038/srep11384 (PMC4466887; doi:10.1038/srep11384)
Supplement: Supplementary Information [file srep11384-s1.pdf]

Title

Morphine compromises bronchial epithelial TLR2/IL17R signaling crosstalk, necessary for lung IL17 homeostasis

Santanu Banerjee*, Jana Ninkovic*, Jingjing Meng#, Umakant Sharma*, Jing Ma*, Richard Charboneau* and Sabita Roy*#.

*Surgery, Basic and Translational Research, University of Minnesota, 515 Delaware St SE, Minneapolis, MN, USA.
#Pharmacology, University of Minnesota, 321 Church St SE, Minneapolis, MN, USA.
Address for Correspondence: Sabita Roy, Professor, Surgery and Pharmacology, 515 Delaware st SE, Minneapolis, MN 55455, Ph: 612-624-4615, FAX: 612-626-4900, email: royxx002@umn.edu.

Supplementary Figure 1

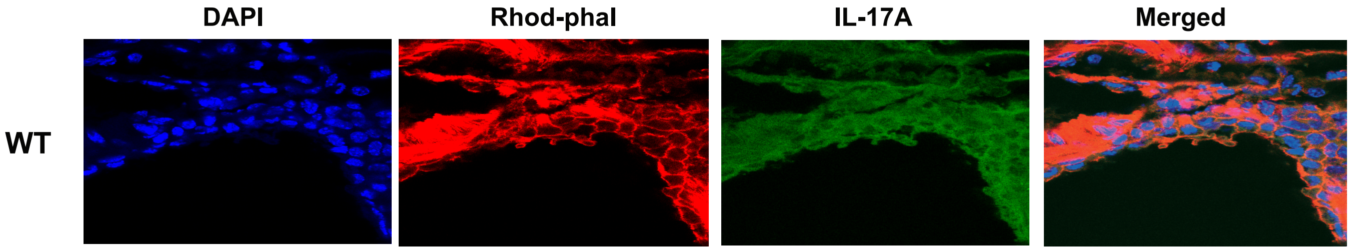

WT animals were treated with Pneomococcal lysate (PL; intra-nasally) for 3hours (see methods) and 5µm sections were obtained using a cryostat (Leica)and stained with FITC conjugated anti-IL17 antibody (eBiosciences). Data shows the bronchial epithelial cells harboring apool of IL17 in the cytoplasm even upon 3hours of PL administration.

Supplementary Figure 2

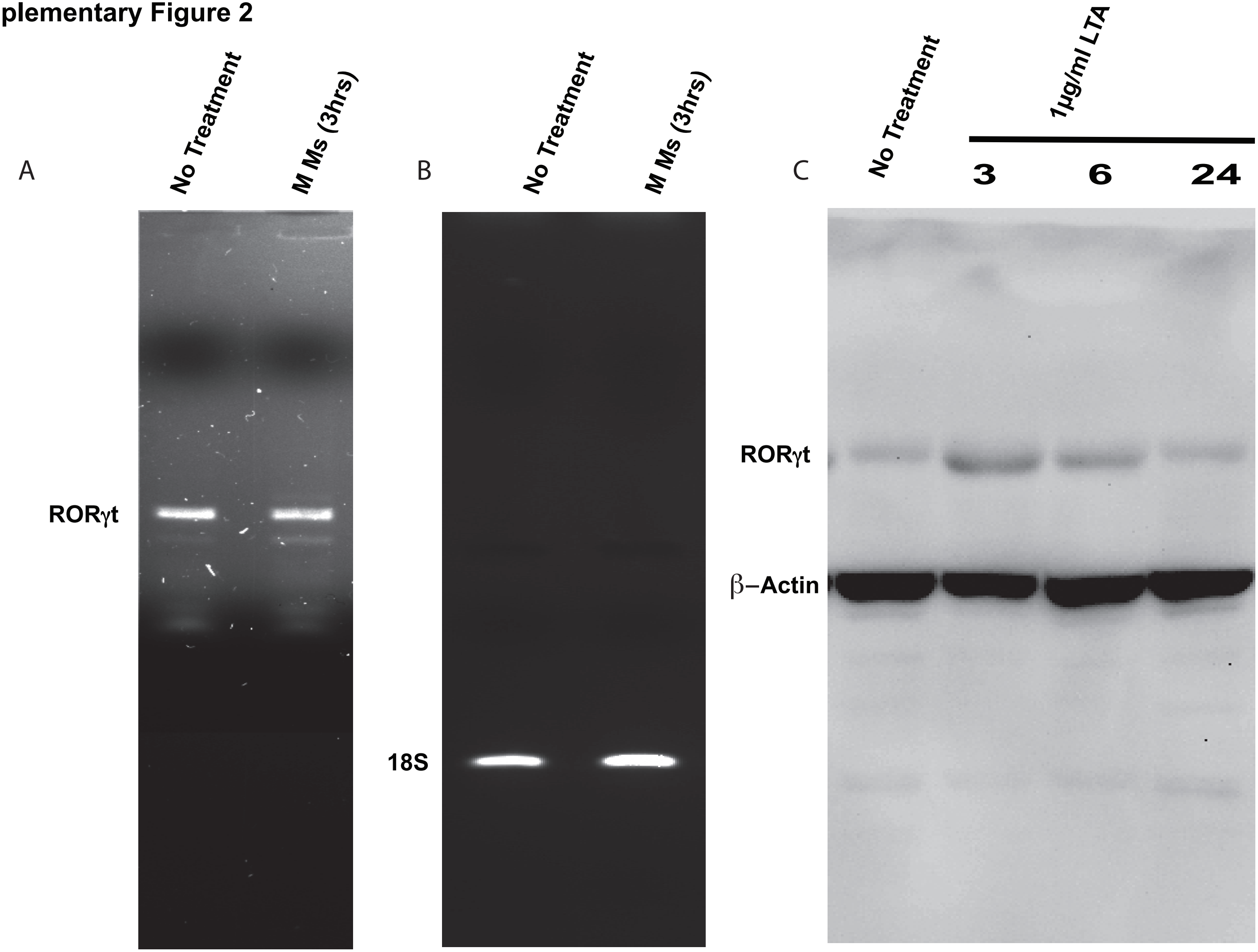

Whole gel profile of RORγt (A) and 18s loading control (B) for Main Figure 2C. Whole membrane western blot profile for RORγt and β-actin (C) for Main Figure 2D.

Supplementary Figure 3

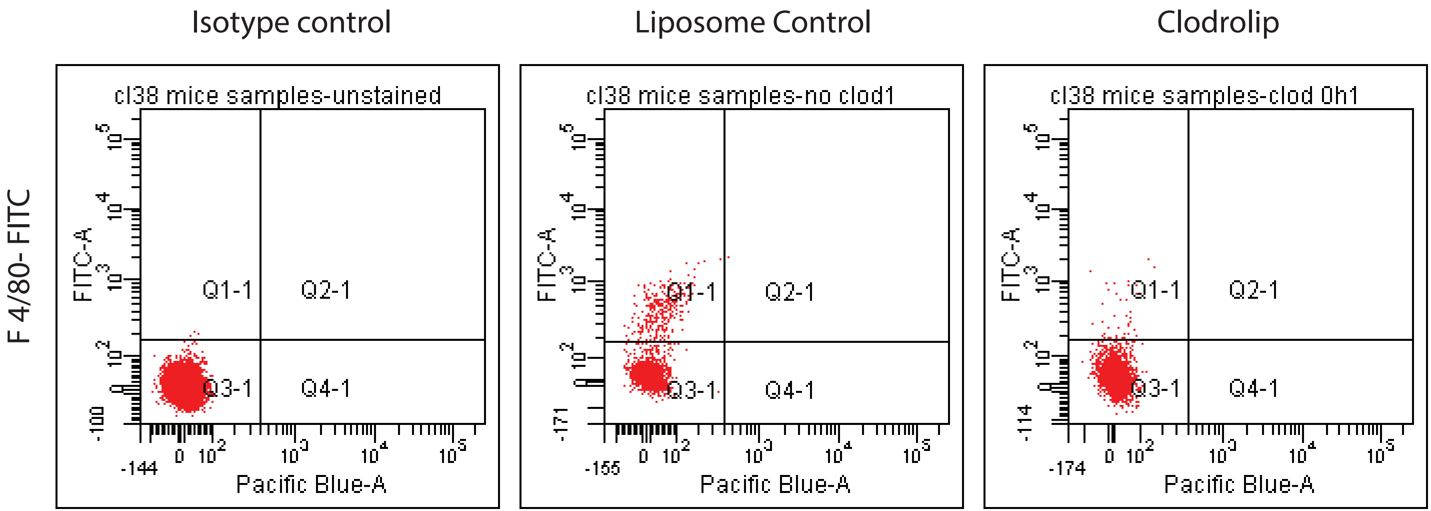

Clodronate (Dichloromethylene Biphosphonic acid), encapsulated in liposomes was purchased from Encapsula nanosciences (Brentwood, TN) along with control empty liposomes. γδ T cells deficient mice (GDKO) were intra-nasally administered with 40μl of the commercial formulation every 48 hours thrice. PL was administered intra-nasally 48 hours after the final clodrolip administration for 3 and 24 hours. At the end of the experiment, efficacy of clodrolip was verified by measuring the depletion of F4/80+ macrophages from the BAL cells using F4/80-V450 (eBiosciences, San Diego, CA) in a BD FACS Canto II flow cytometer.

Supplementary Figure 4

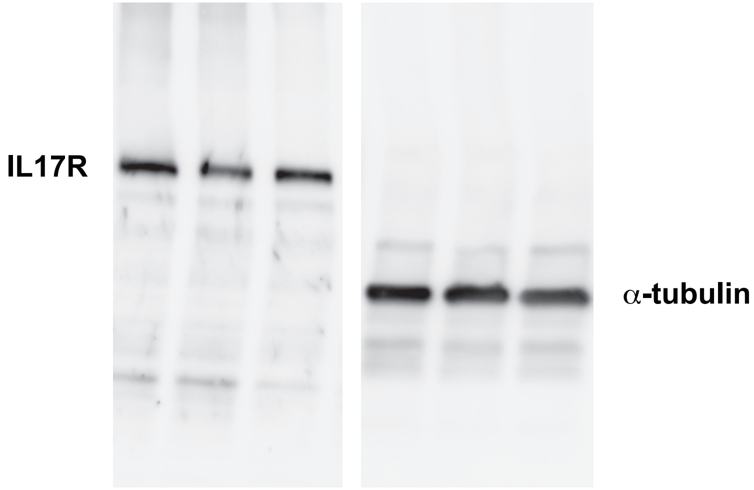

Human Bronchial Epithelial cell line, 16HBE14o was cultured on fibronectin-coated plates (see methods). Cells were counted and equal numbers of cells were lysed directly in SDS PAGE sample buffer and run on a 4-20% ready gel (Bio-Rad, Hercules, CA) and transferred onto a nitrocellulose membrane (Bio-Rad) and probed for IL17RA (antibody from Sigma, St. Louis, MO). The same membrane was also probed with α-tubulin antibody (santa cruz), incubated with IRdye secondary antibodies and the blot developed on Licor Imager.

Supplementary Figure 5

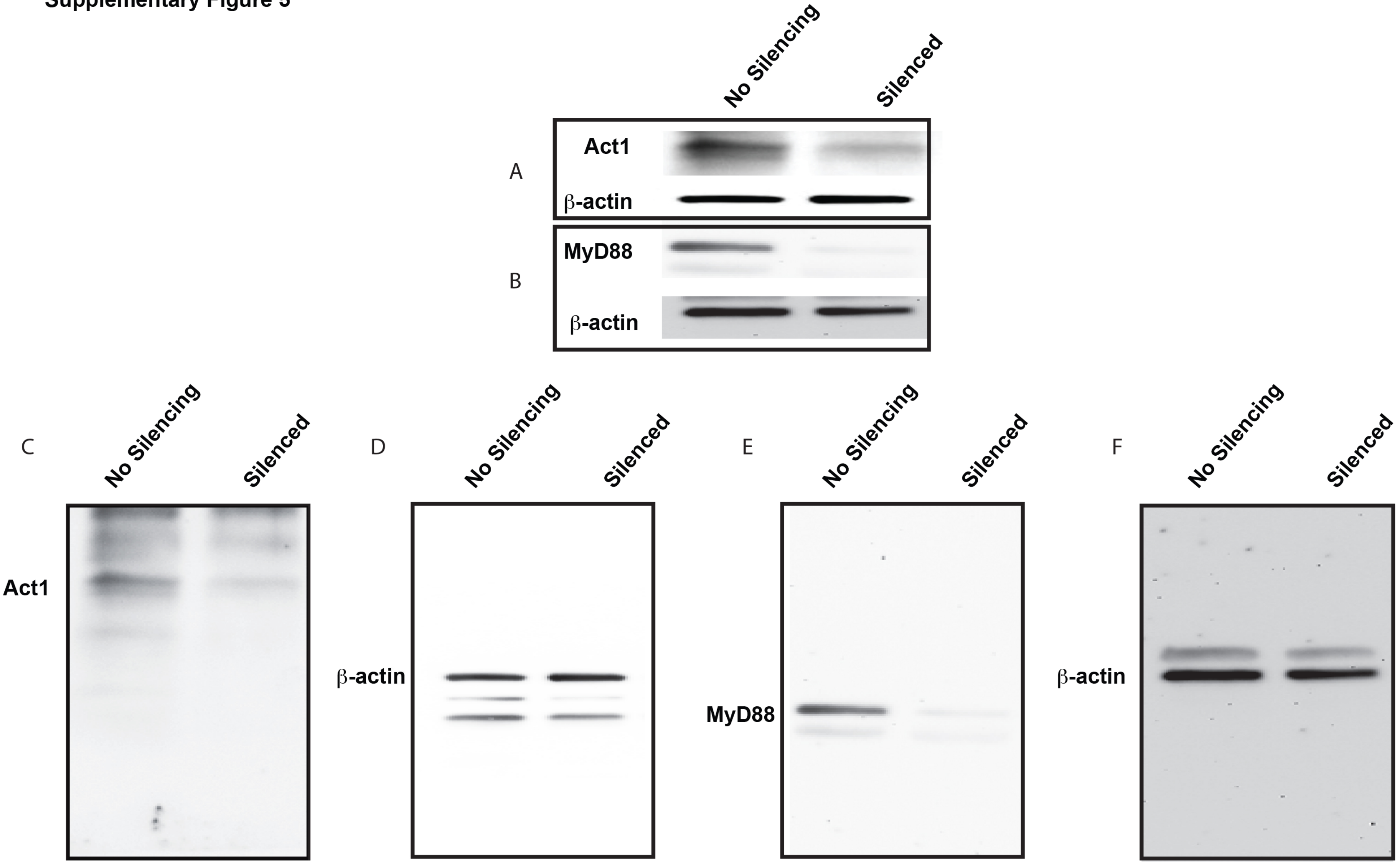

Human bronchial epithelial cell line, 16HBE14o was grown on fibronectin-coated 6 well plates (Corning, NY) to 60-65% confluence and transfected with FlexiTube siRNA premix (Qiagen, Germantown, MD) against MyD88 and Act1/CIKS. Scrambled siRNA was used as a control. The cells were transfected twice within 48 hours. Cells from replicate wells were used for verification of silencing with western blot (All antibodies from Cell Signaling Technologies, Danvers, MA). Figure shows western blot analysis of Act1 (A) and MyD88 (B). Full scan profile of Act1 (A) is represented in (c) and (D), and that of MyD88 (B) is represented in (E) and (F).

Supplementary Figure 6

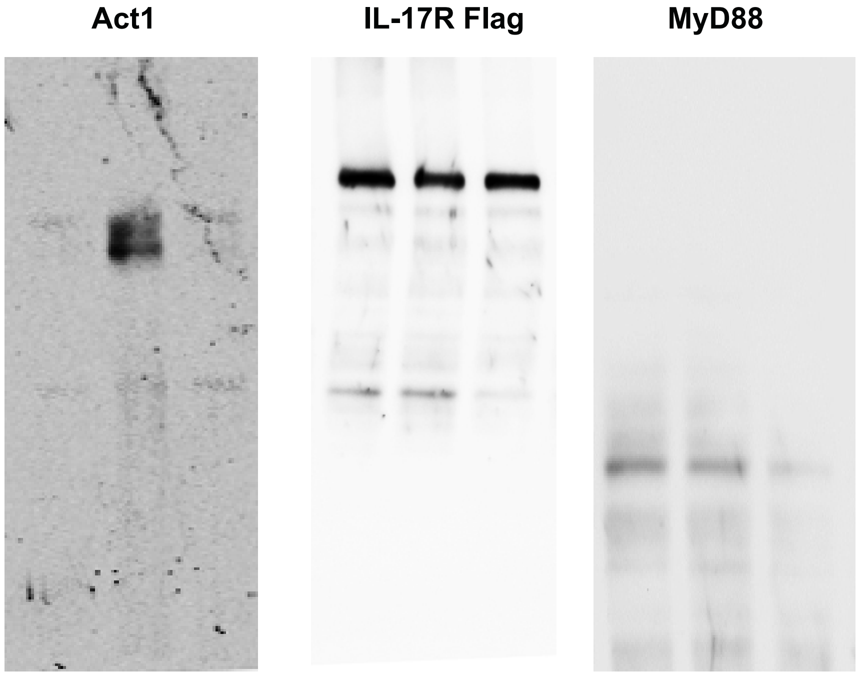

Whole gel Western blot profiles of immune-precipitation experiment described in main Figure 5B

Supplementary Figure 7

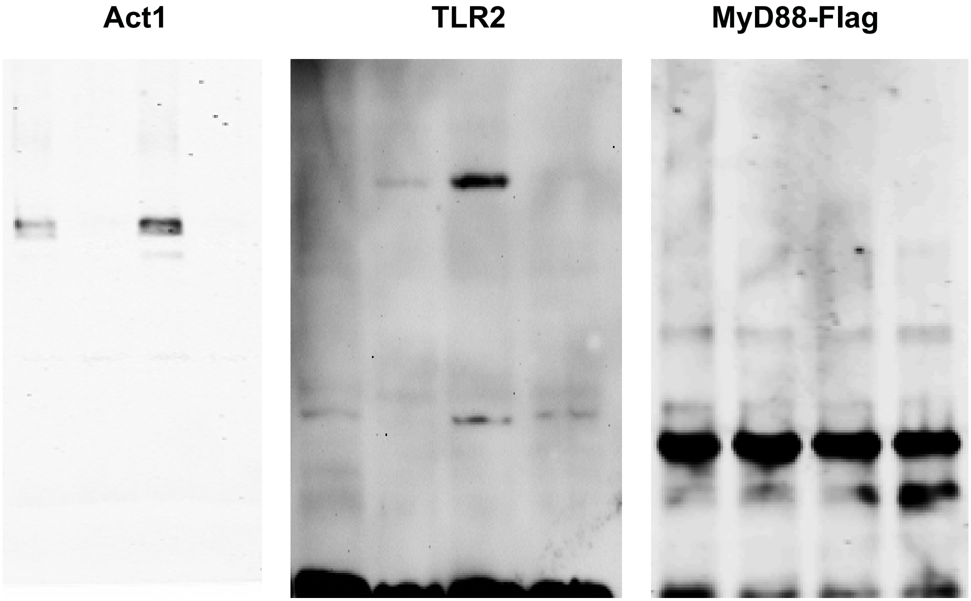

Whole gel Western blot profiles of immune-precipitation experiment described in main Figure 5C

Supplementary Figure 8

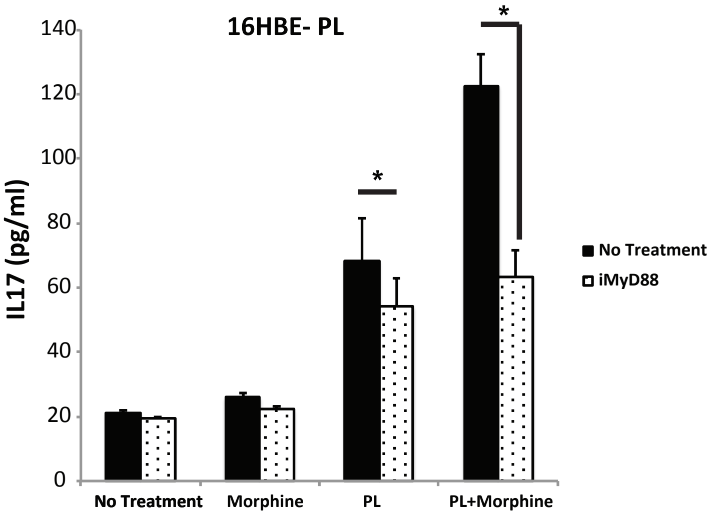

Human Bronchial Epithelial cell-line (16HBE14o) were pre-conditioned with saline/morphine for 24hours and treated with PL for 24 hours. To inhibit MyD88-dependent signaling, a separate set of wells were pretreated with MyD88 homodimerization inhibitory peptide or control peptide (Imgenex) at 100μM for 24 h before in vitro cell treatment with PL. The inhibitory peptide contains a sequence from the MyD88 TIR homodimerization domain, which specifically inhibits the MyD88-dependent pathway (Ma, J., Wang, J., Wan, J., Charboneau, R., Chang, Y., Barke, R. A., and Roy, S. (2010) Infect. Immun. 78, 830–837). Data shows a significant inhibition of IL17 upon Myd88 inhibition with or without morphine; indicating that enhanced release of IL17 at 24 hours is MyD88 dependent [Error bars=SD; *=$p<0.05$].
